# Age‐related loss of hysteresis in the spontaneous vascular sympathetic baroreflex is associated with altered neural rather than mechanical control

**DOI:** 10.1113/EP094070

**Published:** 2026-09-02

**Authors:** Guto Wyn Hughes, Denis J. Wakeham, Christopher J. A. Pugh, Zoe H. Adams, Rachel N. Lord, Jonathan P. Moore

**Affiliations:** ^1^ Institute for Applied Human Physiology, School of Sport Science, College of Medicine and Health Bangor University Bangor Wales UK; ^2^ Department of Internal Medicine, Division of Vascular Medicine The University of Texas Southwestern Medical Centre Dallas Texas USA; ^3^ Centre for Cardiovascular Research, Innovation and Development, Cardiff School of Sport and Health Sciences Cardiff Metropolitan University Cardiff Wales UK; ^4^ School of Life Course and Population Health Sciences Kings College London London UK

**Keywords:** autonomic regulation, baroreflex, cardiovascular control, microneurography, sympathetic nerve activity

## Abstract

The vascular sympathetic baroreflex is critical for stabilising blood pressure. A notable feature of this reflex is hysteresis, a directional asymmetry in responsiveness during rising versus falling pressure that is evident in young adults but attenuated in older age. Whether age‐related reduction reflects altered mechanical transduction at baroreceptors or changes in neural processing remains unclear. We compared the mechanical and neural components of the sympathetic baroreflex, with specific focus on hysteresis, in healthy young (21–30 years old, *n* = 20) and middle‐aged (50–63 years, *n* = 14) males. Continuous recordings of arterial pressure (photoplethysmography), muscle sympathetic nerve activity (MSNA; microneurography) and common carotid artery diameter (ultrasound) were obtained during 6 min of supine rest. Spontaneous baroreflex gain was quantified for mechanical (diastolic pressure into carotid diameter) and neural (carotid diameter into MSNA) components, and overall (diastolic pressure into MSNA), with rising and falling pressure sequences analysed separately. Young men exhibited clear hysteresis in the overall baroreflex loop, whereas this was absent in middle‐aged men. Mechanical hysteresis was not observed in either age group. In contrast, young men displayed pronounced neural hysteresis (Falling: −0.302 ± 0.157 vs. Rising: −0.128 ± 0.88% bursts µm^−1^, *P* < 0.001; *g* = 1.37), whereas neural hysteresis was absent in middle‐aged men (Falling: −0.345 ± 0.400 vs. Rising: −0.396 ± 0.368% burst µm^−1^, *P* = 0.348, *g* = 0.13). These findings suggest that age‐related loss of spontaneous sympathetic baroreflex hysteresis is more apparent within the neural rather than the mechanical component.

## Abstract (highlights)

- **What is the central question of this study?**
  Does the age‐related reduction in sympathetic baroreflex hysteresis reflect differences in mechanical baroreceptor behaviour or in neural control of sympathetic outflow under resting conditions?
- **What is the main finding and its importance?**
  Using a component‐resolved, spontaneous approach in healthy young and middle‐aged men, we show that age‐related attenuation of baroreflex hysteresis occurs despite preserved mechanical sensitivity and is more apparent within the neural than the mechanical component of the reflex arc. These findings are consistent with altered neural regulation of sympathetic outflow during midlife.

## 1 INTRODUCTION

The arterial baroreflex is a fundamental homeostatic mechanism that stabilises blood pressure through beat‐to‐beat adjustments of parasympathetic and sympathetic outflow to the heart and blood vessels (Sagawa, 1983). Studies examining human muscle sympathetic nerve activity (MSNA) at rest provided early evidence that baroreflex control of sympathetic outflow is direction dependent. Notably, Sundlof & Wallin (1978) observed that sympathetic bursts occurred more frequently during transient reductions in arterial pressure than during pressure increases, with burst amplitude also tending to increase as diastolic pressure fell. These findings suggested that sympathetic outflow is more readily recruited during falling than rising arterial pressure, a phenomenon now termed baroreflex hysteresis.

Subsequent studies demonstrated that sympathetic baroreflex can be described as a difference in stimulus–response gain, with gain greater during falling than rising pressure, using both spontaneous fluctuations (Hart et al., 2011) and pharmacologically induced perturbations (Studinger et al., 2009). This asymmetry has been proposed to facilitate rapid sympathoexcitation during hypotensive challenges, compensating for delays in sympathetic and vascular responses, thus providing protection against acute reductions in arterial pressure. One possible explanation is that rising and falling responses operate along distinct stimulus–response relations, with the falling pressure curve displaced relative to the rising curve (Incognito et al., 2020).

Importantly, the magnitude of this hysteresis is attenuated in older compared with young adults (Hart et al., 2011), suggesting an age‐related alteration in the processes governing direction‐dependent baroreflex responsiveness. Whether this attenuation arises primarily from impaired mechanical transduction of the pressure stimulus, altered neural responsiveness or a combination of both remains unclear. However, one prevailing hypothesis has emphasized vascular stiffening in older individuals, which may impair barosensory vessel deformation and consequently blunt sympathoexcitation during pressure falls while accentuating sympathoinhibition during pressure rises (Studinger et al., 2009).

Resolving the mechanisms underlying baroreflex hysteresis benefits from consideration of the component structure of the baroreflex. The mechanical component reflects transduction of arterial pressure at the baroreceptors, whereas the neural component encompasses afferent signalling, central processing and efferent sympathetic output (Taylor et al., 2014). Conventional measures of integrated baroreflex gain reflect the combined interaction of these components without resolving their individual contributions to hysteresis. Furthermore, most studies of sympathetic baroreflex function have focused on MSNA burst occurrence or probability, despite evidence that mechanism governing burst initiation and burst magnitude may be at least partially separable (Kienbaum et al., 2001). Consequently, age‐related differences in hysteresis may be expressed through changes in burst recruitment, burst magnitude or both. Accordingly, it remains unclear whether baroreflex hysteresis arises primarily from the mechanical transduction of pressure at the baroreceptors or from downstream neural processing within the baroreflex arc.

Applying this component framework, Studinger et al. (2009) provided the first detailed separation of the mechanical and neural components of the vascular sympathetic baroreflex using the carotid ultrasound technique developed by Hunt et al. (2001). Using the modified Oxford method, they demonstrated pronounced component‑level hysteresis in young adults, characterised by lower mechanical gain but higher neural gain during pressure falls compared with pressure rises. Compared with young adults, older adults exhibited a uniformly reduced mechanical component gain across both phases of the pressure stimulus, together with an enhanced neural component gain, such that integrated baroreflex gain was preserved. These findings revealed age‑related differences that are not apparent from assessment of integrated gain alone and suggested that ageing may differentially affect the mechanical and neural component of the vascular sympathetic baroreflex. However, the Oxford method probes baroreflex function using pharmacologically induced arterial pressure perturbations that span a wide pressure range but occur over relatively few cardiac cycles. In contrast, spontaneous approaches characterise the baroreflex using several hundred naturally occurring, beat‑to‑beat pressure–MSNA fluctuations within the normal operating range of arterial pressure, trading pressure range for more data points and physiological relevance.

To date, no study has applied spontaneous, beat‑to‑beat approaches to dissociate mechanical and neural components of the sympathetic baroreflex or to examine hysteresis at the component level. Consequently, it remains unclear whether the component‐specific patterns observed during pharmacologically induced pressure perturbations are also evident during spontaneous blood pressure regulation, and whether these mechanisms differ between younger and older individuals. The present study therefore examined the mechanical and neural components of the vascular sympathetic baroreflex, using retrospective analysis of an existing dataset, supplemented by carotid arterial diameter measurements not previously reported, to determine whether age‑related differences in sympathetic baroreflex hysteresis are detectable at the component level in younger and older men at rest. Notably, by studying middle‐aged men, rather than the older populations examined previously, we sought to establish whether alterations in the mechanical and neural determinants of hysteresis emerge earlier in the ageing process. By extending component analysis to many small physiological fluctuations, this study directly tested whether mechanistic insights derived from pharmacological perturbations extend to resting sympathetic blood pressure regulation in young and middle‑aged men.

## 2 METHODS

### 2.1 Ethical approval

All study procedures were approved by the Cardiff School of Sport and Health Sciences Research Ethics Committee (16/7/02R) and conducted in accordance with the *Declaration of Helsinki* (2013), except for registration in a database. Written informed consent was obtained from all participants.

### 2.2 Participants and study design

Seventy men from the local community were screened for participation in this study. Those participants enrolled into the study represented a wide age range (21–63 years) and a broad spectrum of physical activity levels, from recreationally active individuals (∼ 3 h week^−1^ of structured exercise) to competitive runners (∼4.5 to 9+ h week^−1^ of training). All participants were non‐smokers, normotensive, had a BMI <30 kg m^−2^, and reported no history of cardiovascular, respiratory, metabolic or neurological disease. The data presented herein were derived from a subset of a cohort described previously (Wakeham et al., 2019). Consistent with several other studies from this cohort (Lord et al., 2020; Talbot et al., 2020; Wakeham et al., 2022), participants were stratified into young and middle‐aged groups to facilitate examination of age‐related differences in vascular sympathetic hysteresis and the relative contributions of mechanical and neural factors. Therefore, training status was not considered as a separate variable in the present analyses. This approach was supported by previous findings from the parent cohort demonstrating limited effects of training status on autonomic variables relevant to the present study, with no differences in MSNA burst frequency or vascular sympathetic baroreflex gain observed between runners and non‐runners within either age group (Wakeham et al., 2019).

### 2.3 Experimental procedures and measurements

#### 2.3.1 Visit 1: Screening and assessment of cardiorespiratory fitness

Participants attended the Exercise Physiology Laboratory (∼22°C) at Cardiff Metropolitan University having consumed a light meal at least 2 h prior to the testing visit. Height (Holtain Stadiometer, Holtain Ltd, Crosswell, UK), body mass (SECA 770 scale, Vogel & Halke, Hamburg, Germany) and resting blood pressure (manual sphygmomanometer, Welch Allyn, Aylesbury, UK) were measured to confirm eligibility (BMI <30 kg m^−2^; resting blood pressure <140/90 mmHg). Participants reported prescription and over‐the‐counter medication use and were excluded if taking any medication at the time of testing. Cardiorespiratory fitness was assessed using an incremental exercise test to volitional exhaustion on a cycle ergometer (Lode Corival, Groningen, The Netherlands) with work rate increased by 20 W min^−1^ as previously described (Wakeham et al., 2019).

#### 2.3.2 Visit 2: Experimental testing

Participants attended the laboratory on a separate day following a ≥6 h fast and after abstaining from caffeine, alcohol and strenuous exercise for at least 24 h. Following instrumentation and identification of a stable MSNA recording site, participants rested supine for at least 10 min, during which cardiac echocardiography was performed. Participants then completed a 6‐min assessment of sympathetic vasomotor outflow and spontaneous sympathetic baroreflex function. Participants breathed freely (i.e. without paced or controlled breathing), while continuous recordings of arterial pressure, heart rate, muscle sympathetic nerve activity (MSNA) and right common carotid artery diameter were acquired to enable time‑aligned, beat‑by‑beat assessment of baroreflex‑relevant variables.

### 2.4 Haemodynamics and resting sympathetic neural activity

Heart rate and blood pressure were monitored continuously via three‐lead electrocardiography and finger photoplethysmography (FinometerPro, FMS, Groningen, The Netherlands). Multiunit MSNA was recorded from the common peroneal nerve by an experienced microneurographer (J.P.M.). A tungsten microelectrode (200 µm diameter, 50 mm long, tapered to a 1‐ to 5‐µm uninsulated tip; FHC, Bowdoin, ME, USA) was inserted percutaneously into the nerve of the left or right leg at the popliteal fossa. A second uncoated tungsten reference electrode was inserted subcutaneously 1–3 cm from the recording site. The recorded neurogram was amplified (70,000–160,000‐fold), band‑pass filtered (band width 700–2000 Hz), full‐wave rectified and integrated with a resistance–capacitance circuit (decay constant 0.1s) using a Nerve Traffic Analyzer (model 662C‐3, Iowa University Bioengineering, University of Iowa, Iowa City, IA, USA). A valid recording site was confirmed by the presence of pulse‑synchronous bursts, increased activity during end‑expiratory breath hold, absence of response to a startle stimulus, and 3:1 signal to noise ratio (White et al., 2015). Following identification of an acceptable MSNA signal, participants rested for at least 10 min to enable signal stabilization prior to data acquisition. No adverse events or complications occurred during or following the microneurography procedure in any subject.

### 2.5 Ultrasound procedures

Echocardiograms were acquired using a commercially available ultrasound system (Vivid E9, GE Healthcare, Horten, Norway) with a 4 MHz array probe. Images were obtained from apical four‐ and two‐chamber views of the left ventricle by a single experienced sonographer (R.N.L.) and saved for offline analysis, with commercially available software (EchoPac BT13, GE Healthcare). Stroke volume (SV) was calculated using Simpson's biplane method, allowing for the calculation of cardiac output (${\dot{Q}}_{c}$: HR × SV) and total peripheral resistance (TPR: ${\dot{Q}}_{c}$/mean arterial pressure).

Longitudinal B‐mode images of the right common carotid artery (CCA) were acquired by an experienced vascular sonographer (C.J.P.) using a 12‑MHz linear array transducer (Vivid Q, GE Healthcare), positioned approximately 1–2 cm proximal to the carotid bulb at a 60° insonation angle. The ultrasound output was connected to a video‐capture device (VGA2USB, Epiphan Systems Video, Ottawa, ON, Canada) and images were recorded using Camtasia® video‑capture software (TechSmith Corp., East Lansing, MI, USA) at a frame rate of 30 frames s^−1^ and a resolution of 800 × 600 (SVGA). All recordings were saved in digital format and analysed offline.

### 2.6 Data analysis

Haemodynamic and MSNA data were sampled at 1000 Hz using commercially obtainable acquisition software (LabChart Pro v8.3.1, ADInstruments, Bella Vista, Australia). Finger photoplethysmography derived blood pressure waveforms were calibrated at regular intervals to systolic and diastolic pressures measured via manual sphygmomanometry. Systolic blood pressure (SBP), diastolic blood pressure (DBP) and mean arterial pressure (MAP), determined from maximum, minimum and mean values, were calculated on a beat‐by‐beat basis from the finger arterial pressure waveform.

Multiunit bursts of MSNA were identified and verified by visual inspection by a single investigator (G.W.H.) following adjustment for baroreflex latency (i.e., the interval between ECG R wave and peak burst height), which aligned each burst with the appropriate R wave of the ECG (White et al., 2015). MSNA was quantified as burst frequency (burst min^−1^), burst incidence (burst 100 heartbeats^−1^) and average burst area (modulus of the integrated neurogram ± 0.4 s around an identified burst, after normalising the neurogram to the tallest burst). Burst area reflects burst strength, burst frequency reflects the amount of sympathetic vasoconstrictor outflow and associated neurotransmitter release, and burst incidence provides a time‐independent measure baroreflex ‘gating’ of sympathetic vasoconstrictor activity (Charkoudian & Wallin, 2014).

Beat‑to‑beat CCA diameter was analysed using commercially available software (Carotid Analyser, Medical Imaging Applications LLC, Coralville IA, USA), with the ultrasound recordings processed using frame‑by‑frame edge‑detection. Following diameter extraction, the beat‑to‑beat CCA waveform was exported to LabChart and synchronized with simultaneously recorded haemodynamics and MSNA signals using LabChart's time‑alignment tools. This procedure ensured that carotid diameter, arterial pressure and sympathetic neural activity were appropriately matched on a beat‐to‐beat basis for subsequent analysis of the neural and mechanical components of the baroreflex.

Beat‐by‐beat data were exported from LabChart and imported into an analysis package, (Spike2, version 7.14, Cambridge Electronic Design, Cambridge, UK) using a custom‐built script. DBP values were averaged into 3 mmHg bins to reduce variability arising from non‐baroreflex mechanisms (e.g., respiration). CCA diameter for the neural and mechanical components was taken as the average diameter of each associated 3 mmHg DBP bin. Spontaneous sympathetic baroreflex sensitivity was calculated as the slope (gain) of the linear regression for each of the following stimulus–response relationships.

1. Mechanical component: DBP into CCA diameter.
2. Neural component: CCA diameter into MSNA (burst probability, burst area).
3. Integrated component: DBP into MSNA (burst probability, burst area).

We completed the analysis on all DBP values over the full 6 min of data collection to determine overall baroreflex gain, which was not dependent on whether DBP was increasing or decreasing (this reflected overall baroreflex sensitivity). We then completed the same analysis during spontaneous increases or decreases in DBP. More specifically, to calculate spontaneous baroreflex sensitivity to rising arterial pressure, cardiac cycles preceded by a lower DBP were used. For spontaneous sympathetic baroreflex sensitivity to falling arterial pressure, we used cardiac cycles that were preceded by cardiac cycles with a higher DBP. This meant that two total cardiac cycles were included for each segment. To reduce the influence of very small beat‐to‐beat variation and measurement error on directional classification, only cycle pairs with an absolute DBP difference of ≥0.5 mmHg were included in the analysis. All linear regression analyses were weighted by the number of cardiac cycles contributing to each DBP bin. Subjects whose overall integrated or neural burst probability correlation coefficients were below 0.7 were excluded, in accordance with quality criteria described by Holwerda et al. (2021). No additional exclusion criteria were applied after the separating rising and falling pressure sensitivities.

### 2.7 Statistical analyses

All data are presented as means ± SD. Normality was assessed using the Shapiro–Wilk test. Descriptive and baseline characteristics were grouped by family (anthropometrics, haemodynamics, MSNA indices, carotid artery diameters) and compared between groups using multiple unpaired Student's *t*‐tests with Welch's correction (individual variance for each group) and correction for multiple comparisons using the Holm–Šidák method. Sympathetic baroreflex sensitivities derived from the rising‑pressure and falling‑pressure domains were compared using two‑way repeated measures ANOVA (factors: age group × pressure direction) with *post hoc* comparisons conducted using Fisher's LSD. Overall sympathetic baroreflex sensitivities were compared between groups using unpaired *t*‐tests (integrated burst probability, integrated burst area, mechanical component) or Mann–Whitney tests (neural burst probability, neural burst area). Effect sizes were quantified for all variables other than participant demographics using Hedges’ *g* (small: 0.20; medium: 0.5; large: 0.8), which provides an unbiased estimate of standardized mean differences. Statistical significance was accepted at *P* < 0.05. Analyses were conducted using GraphPad Prism (Version 11.0.2; GraphPad Software, Boston, MA, USA).

## 3 RESULTS

Simultaneous recordings of haemodynamic variables, muscle sympathetic nerve activity and carotid artery ultrasound images were acquired for 20 young men and 17 middle‑aged men. However, data from three middle‑aged participants did not meet predefined criteria for assessment of sympathetic baroreflex gain and were therefore removed from subsequent analysis, resulting in *n* = 14 for this group.

### 3.1 Participant characteristics, resting haemodynamics, basal sympathetic neural activity and carotid arterial structure

Table 1 summarizes participant characteristics and resting blood pressure. As expected, young participants were younger than middle‑aged. Height, body mass and BMI were not different. Although differences in systolic, diastolic and mean arterial pressures showed moderate‑to‑large effect sizes (Hedges’ *g* = 0.61–1.06), only systolic pressure reached significance (*P* = 0.041), while mean pressure (*P* = 0.058) and diastolic pressure (*P* = 0.132) did not. Resting heart rate did not differ between age groups. The effect size for stroke volume was large (*g* = 0.87) but did not reach significance (*P* = 0.058). Cardiac output was larger in the younger participants (*P* = 0.015) and total peripheral resistance was higher in the middle‐aged (*P* < 0.001). Cardiorespiratory fitness was not different between groups (*P* = 0.203).

**TABLE 1:** Participant characteristics and haemodynamic profiles in young and middle‑aged adults.

	Young (*n* = 20)	Middle‐aged (*n* = 14)	*P* (*t*‐test)	Effect size (*g*)
Age (years)	22 ± 3	54 ± 4	<0.001	
Height (cm)	179.1 ± 5.0	176.8 ± 7.2	0.661	
Body mass (kg)	72.5 ± 13.9	72.8 ± 9.8	0.942	
BMI (kg/m^2^)	22.7 ± 4.1	23.3 ± 2.1	0.821	
SBP (mmHg)	117 ± 9	127 ± 10	0.041	1.02
DBP (mmHg)	72 ± 9	77 ± 7	0.132	0.62
MAP (mmHg)	87 ± 8	94 ± 7	0.058	0.87
Heart rate (bpm)	51 ± 11	48 ± 10	0.426	0.27
SV (mL)	80 ± 17	67 ± 13	0.058	0.87
${\dot{Q}}_{c}$ (L min^−1^)	4.01 ± 0.52	3.29 ± 0.63	0.015	1.26
TPR (mmHg L min^−1^)	22.22 ± 3.73	29.24 ± 4.55	<0.001	1.72
${\dot{V}}_{O_{2}\mathrm{peak}}$ (ml kg^−1^ min^−1^)	50.4 ± 15.2	44.5 ± 11.1	0.203	0.43

*Note*: Values are means ± SD. Group differences were assessed using independent‑samples tests, with *P*‑values presented for all variables and Hedges’ *g* effect sizes reported where applicable. Anthropometric measures include height, body mass and BMI (body mass index). Haemodynamic variables include resting systolic blood pressure (SBP), diastolic blood pressure (DBP), and mean arterial pressure (MAP), heart rate, stroke volume (SV), cardiac output (${\dot{Q}}_{c}$) and total peripheral resistance (TPR). Cardiorespiratory fitness is shown as peak oxygen consumption (${\dot{V}}_{O_{2}\mathrm{peak}}$).

Table 2 summarizes basal MSNA and carotid arterial diameter characteristics. Middle‑aged men demonstrated significantly higher basal MSNA than young men. Burst frequency was nearly doubled (31 ± 7 vs. 16 ± 8 bursts min^−1^, *P* < 0.001; *g* = 1.98) and burst incidence – the operating point for spontaneous vascular sympathetic baroreflex function – was similarly elevated (66 ± 16 vs. 32 ± 19 bursts 100 beats^−1^, *P* < 0.001; *g* = 1.88). Mean burst area did not differ between groups (*P* = 0.707). As expected, middle‑aged men had significantly larger common carotid artery peak‐systolic (*P* = 0.017) and end‐diastolic (*P* = 0.002) diameters as well as lower absolute carotid distension (*P* = 0.017).

**TABLE 2:** Muscle sympathetic nerve activity (MSNA) and carotid arterial diameter characteristics in young and middle‑aged adults.

	Young (*n* = 20)	Middle‐aged (*n* = 14)	*P* (*t*‐test)	Effect size (*g*)
MSNA				
Bursts min^−1^	16 ± 8	31 ± 7	<0.001	1.98
Bursts 100 beats^−1^	32 ± 19	66 ± 16	<0.001	1.88
Burst area (%)	15.3 ± 1.8	15.7 ± 3.4	0.707	0.15
Carotid arterial diameter				
Peak‐systolic (mm)	7.18 ± 0.47	7.61 ± 0.46	0.017	0.94
End‐diastolic (mm)	6.60 ± 0.50	7.16 ± 0.37	0.002	1.24
Distension (mm)	0.58 ± 0.11	0.45 ± 0.14	0.017	1.04

*Note*: Values are means ± SD. Group comparisons were performed using independent‐samples tests, with corresponding *P*‐values and Hedges’ *g* effect sizes reported. Bursts min^−1^ and bursts 100 beats^−1^ represent indices of MSNA burst frequency and burst area (%) reflects integrated burst strength. Carotid arterial measures include peak‐systolic and end‐diastolic diameters, as well as arterial distension (peak‐systolic minus end‐diastolic diameter).

### 3.2 Vascular sympathetic baroreflex

**FIGURE 1:**
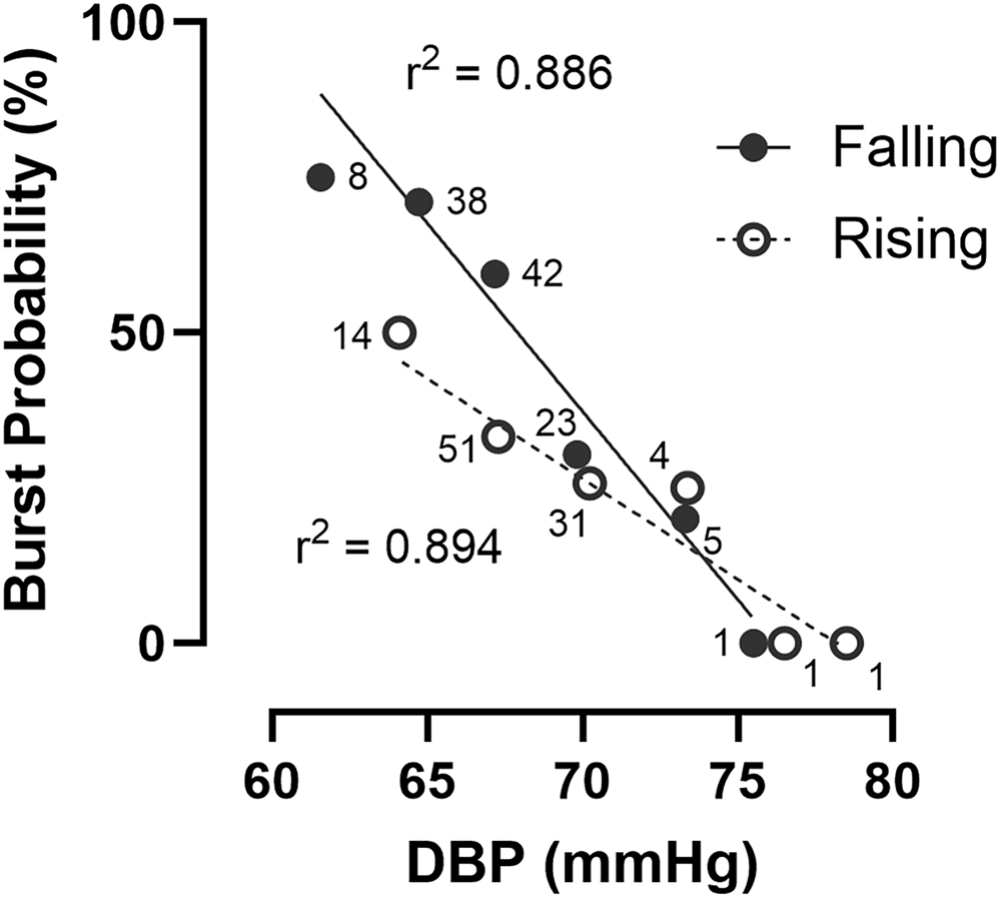
Integrated baroreflex sensitivity slopes separated by responses to falling and rising pressure in a young male participant. Continuous lines represent data obtained during falling pressure; dashed lines represent data obtained during rising pressure. Numbers adjacent to each point indicate the number of cardiac cycles contributing to each pressure bin. Coefficients of determination (*r* ^2^) for each relationship are also shown.

**FIGURE 2:**
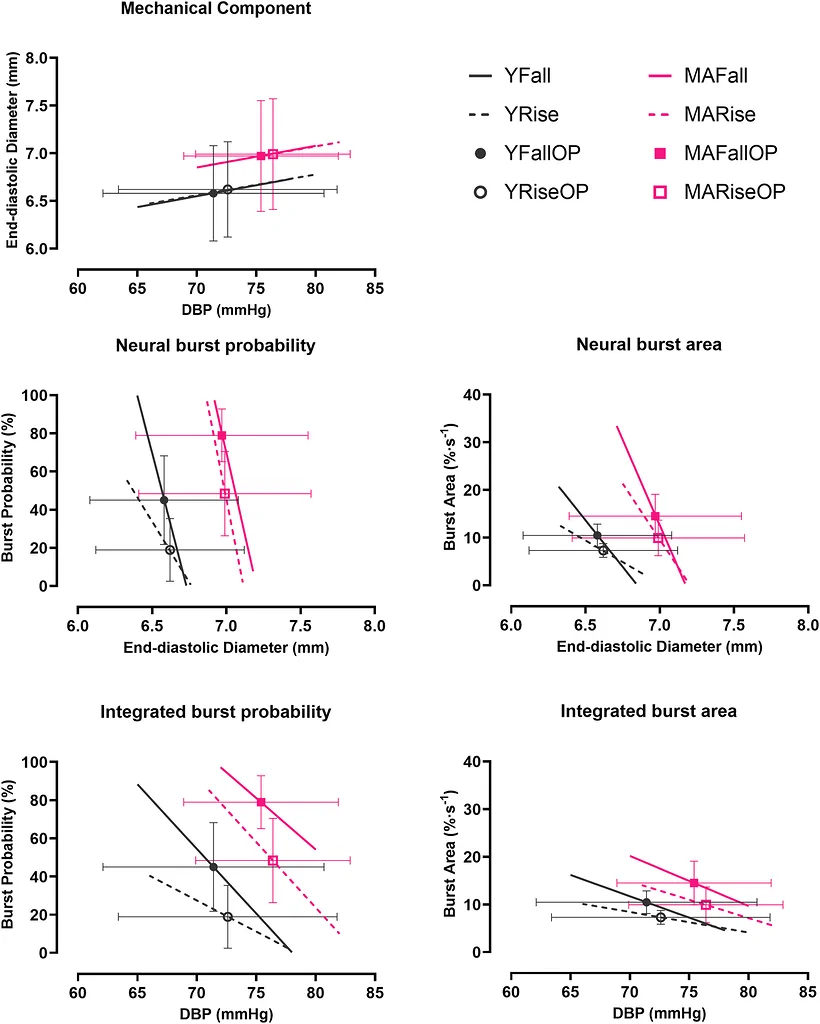
Hysteresis characteristics of mechanical, neural and integrated baroreflex in young and middle‐aged men. Continuous lines represent data obtained during falling pressure; dashed lines represent data obtained during rising pressure. Component operating points (means ± SD) are superimposed on the regression lines as circles in young men and squares for middle‐aged men. YFall, young falling pressure gain; YRise, young rising pressure gain; YFallOP, young falling pressure operating point; YRiseOP, young rising pressure operating point; MAFall, middle‐aged falling pressure gain; MARise, middle‐aged rising pressure gain; MAFallOP, middle‐aged falling pressure operating point; MARiseOP, middle‐aged rising pressure operating point.

**FIGURE 3:**
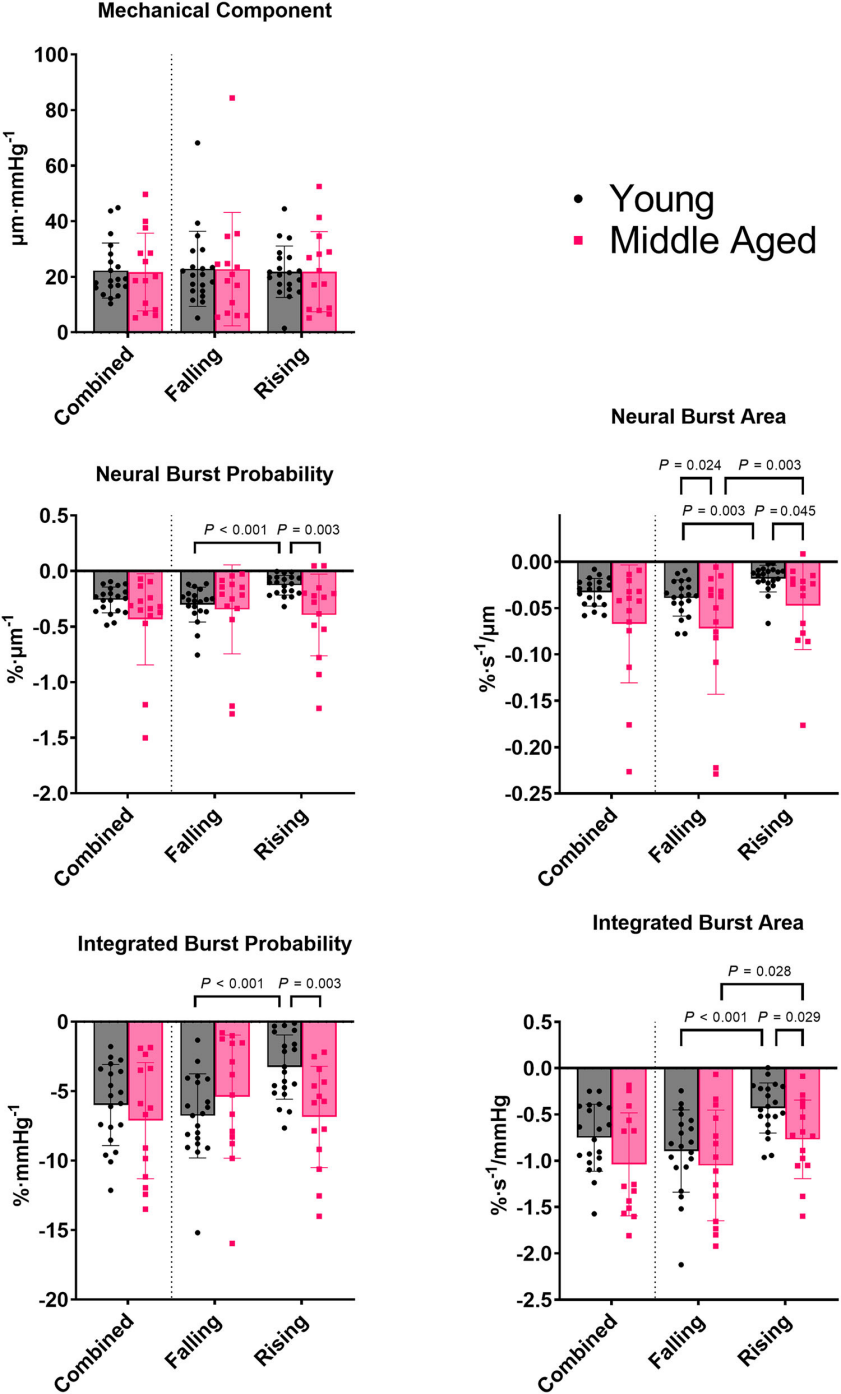
Mechanical, neural, and integrated baroreflex gains derived from spontaneous arterial pressure fluctuations. Mechanical (pressure–diameter), neural (diameter–sympathetic outflow) and integrated baroreflex (pressure–sympathetic outflow) gains are shown for the full baroreflex loop and separately for the falling and rising pressure limbs. Individual values and group means ± SD are presented to illustrate variability and limb‑dependent differences in baroreflex behaviour. Direction dependent response comparisons were performed using two‑way ANOVA (factors: age group × pressure direction). Full baroreflex loop age comparisons were performed using unpaired *t*‐tests on the integrated burst probability, integrated burst area and mechanical components, and Mann–Whitney tests on the neural burst probability and neural burst area components.

Figure 1 provides an example of the separation of integrated baroreflex into distinct falling and rising pressures gains in an individual young male. The number of cardiac cycles in each bin is noted next to each data point. Figure 2 illustrates the hysteresis characteristics of the mechanical and neural components, together with the resultant integrated baroreflex gain, at a group level in young and middle‑aged males. The relationships shown represent the group mean linear regressions of the corresponding stimulus–response functions and respective operating points. Figure 3 provides a detailed quantification of vascular sympathetic baroreflex gain, delineating the mechanical, neural and integrated components across the full baroreflex loop, including separate assessments of the falling and rising pressure.

### 3.3 Mechanical component and direction‑dependent mechanical gain

Overall mechanical gain did not differ significantly between young and middle‑aged men (Young: 22 ± 10 vs. Middle‐aged: 22 ± 14 µm mmHg^−1^, *P* = 0.898). When separated by direction dependant response there was no age × direction interaction (*F*_1,32 _= 0.00, *P* = 0.970), nor a significant main effect of age (*F*_1,32 _= 0.00, *P* = 0.991) or direction (*F*_1,32 _= 0.27, *P* = 0.608) for mechanical gain.

### 3.4 Neural component and direction‑dependent burst‑probability/burst‑area gain

Overall neural burst probability gain was lower in young men compared with middle‐aged, but this did not reach statistical significance (Young: −0.260 ± 0.117 vs. Middle‐aged: −0.435 ± 0.410% bursts µm^−1^, *P* = 0.231, *g* = 0.63). There was no main effect of age (*F*_1,32 _= 3.34, *P* = 0.077) and no main effect of pressure direction (*F*_1,32 _= 3.17, *P* = 0.084). However, there was a significant age × direction interaction (*F*_1,32 _= 10.52, *P* = 0.003). Young men displayed marked hysteresis, with a significantly higher gain during falling pressure compared with rising pressure (falling: −0.302 ± 0.157 vs. rising: −0.128 ± 0.88% bursts µm^−1^, *P* < 0.001; *g* = 1.37). Middle‑aged men showed no such hysteresis (Falling: −0.345 ± 0.400 vs. Rising: −0.396 ± 0.368% burst µm^−1^, *P* = 0.348, *g* = 0.13). As such, rising pressure neural burst probability gain was significantly greater in middle‑aged than young men (*P* = 0.005; *g* = 1.10).

The effect size for overall neural burst area gain was large but did not reach significance (Young: −0.033 ± 0.015 vs. Middle‐aged: −0.067 ± 0.064% s^−1^ µm^−1^, *P* = 0.104; *g* = 0.81). There was a significant main effect of age (*F*_1,32 _= 5.41, *P* = 0.027) and a significant main effect of pressure direction (*F*_1,32 _= 20.51, *P* < 0.001) but no age × direction interaction (*F*_1,32 _= 0.14, *P* = 0.711). Both age groups exhibited greater falling pressure gain than rising‑pressure gain (Young: *P* = 0.003, *g* = 1.23; Middle‑aged: *P* = 0.003, *g* = 0.41). Falling pressure gain was significantly larger in middle‑aged compared with young men (Young: −0.039 ± 0.019 vs. Middle‐aged: −0.072 ± 0.071% s^−1^ µm^−1^, *P* = 0.024; *g* = 0.69), as was rising pressure gain (Young: 0.018 ± 0.014 vs. Middle‐aged: ‐0.047 ± 0.048% s^−1^ µm^−1^, *P* = 0.045; *g* = 0.90).

### 3.5 Integrated component and direction‑dependent baroreflex gain

Overall integrated burst probability gain did not differ between groups (Young: −6.00 ± 2.92 vs. Middle‐aged: −7.12 ± 4.18% bursts mmHg^−1^, *P* = 0.362; *g* = 0.32). There was no main effect of age (*F*_1,32 _= 1.22, *P* = 0.277) and no main effect of pressure direction (*F*_1,32 _= 3.08, *P* = 0.089). However, there was a significant age × direction interaction (*F*_1,32 _= 18.13, *P* < 0.001). Young men exhibit clear hysteresis, with greater falling pressure gain than rising pressure gain (Falling: −6.78 ± 3.03 vs. Rising: −3.26 ± 2.32% bursts mmHg^−1^, *P* < 0.001; *g* = 1.30). Middle‑aged men did not display hysteresis (Falling: −5.39 ± 4.43 vs. Rising: −6.85 ± 3.65% bursts mmHg^−1^, *P* = 0.112; *g* = 0.36). Rising pressure integrated burst probability gain was significantly greater in middle‑aged compared with young men (Young: −3.26 ± 2.32 vs. Middle‐aged: −6.85 ± 3.65% bursts mmHg^−1^, *P* = 0.003; *g* = 1.22), while falling burst probability gain did not differ (Young: −6.78 ± 3.03 vs. Middle‐aged: −5.39 ± 4.43% bursts mmHg^−1^, *P* = 0.236; *g* = 0.38).

Overall integrated burst area gain did not differ significantly between groups but showed a medium effect size (Young: −0.75 ± 0.36 vs. Middle‐aged: −1.04 ± 0.56% s^−1^ mmHg^−1^, *P* = 0.076; *g* = 0.64). There was a significant main effect of pressure direction (*F*_1,32 _= 21.95, *P* < 0.001) but no main effect of age (*F*_1,32 _= 3.66, *P* = 0.065) or age × direction interaction (*F*_1,32 _= 1.33, *P* = 0.258). Young men demonstrated pronounced hysteresis (Falling: −0.89 ± 0.44 vs. Rising: −0.43 ± 0.27% s^−1^ mmHg^−1^, *P* < 0.001; *g* = 1.26). Middle‑aged men also displayed a fall‑to‑rise difference (Falling: −1.05 ± 0.60 vs. Rising: −0.77 ± 0.42% s^−1^ mmHg^−1^, *P* = 0.028; *g* = 0.54), though the magnitude was smaller.

## 4 DISCUSSION

The present study examined age‑related differences in the mechanical and neural components of the vascular sympathetic baroreflex, with particular emphasis on directional behaviour during spontaneously rising and falling pressures. Three principal findings emerged. First, mechanical gain was not different between young and middle‐aged men. Second, the pronounced hysteresis in burst probability gain observed in young men was absent in middle‑aged men. Although overall burst probability gain was not different, the directional asymmetry characteristic of hysteresis was absent. Third, burst area hysteresis was evident within both the integrated and neural components in young and middle‐aged men. Collectively, these findings suggest that the age‑related attenuation of spontaneous sympathetic baroreflex hysteresis is more apparent within the neural than the mechanical component of the reflex arc. However, the specific physiological mechanisms underlying this observation cannot be determined from the present study.

### 4.1 Mechanical preservation and differences in burst initiation

In contrast to findings obtained using the modified Oxford method (Studinger et al., 2009), the present data do not support a mechanical‐deficit model to explain the age‐related attenuation or loss of vascular sympathetic baroreflex hysteresis under physiological conditions. During spontaneous pressure fluctuations, mechanical hysteresis was not evident in either age group and mechanical gain did not differ between young and middle‐aged men. Notably, studies using the larger parent cohort from which this sample was drawn reported greater carotid artery stiffness in middle‐aged men (Talbot et al., 2020; Wakeham et al., 2019). The absence of age‐related differences in mechanical gain in the present study suggests that these structural vascular changes do not necessarily translate into altered dynamic barosensory transduction at rest. Instead, differences emerged in direction‐dependent burst initiation. Young men demonstrated clear hysteresis in sympathetic burst recruitment, characterized by greater burst probability gain during falling pressure, a pattern thought to facilitate rapid sympathoexcitation and protect against transient reductions in arterial pressure. By contrast, middle‐aged men showed convergence of burst probability gain across rising and falling pressure, indicating a loss of hysteresis in burst initiation.

### 4.2 Role of burst strength in neural adaptation

Most studies of sympathetic baroreflex function have focused on MSNA burst incidence or probability. As a result, mechanisms governing burst strength have received comparatively little attention. However, the present findings suggest the regulation of burst strength may provide additional physiological insight. Consistent with the proposal of Kienbaum et al. (2001), burst initiation and discharge strength may be regulated by partially distinct processes within the baroreflex arc, potentially contributing to the differential hysteresis observed in burst probability and burst area.

By analysing the relationship between changes in arterial pressure and burst area, the present study captured regulatory features that would be overlooked if burst incidence alone were considered. Although sympathetic baroreflex hysteresis has been described previously using indices of burst occurrence, the directional dependence of the burst strength–pressure relationship has received little attention and, to our knowledge, has not been systematically quantified. Burst area hysteresis was evident in both young and middle‐aged men. Moreover, both the rising and falling burst area neural component gains were greater in the middle‐aged men. These findings suggest that preserved mechanical gain is accompanied by altered directional weighting in burst initiation, but not of burst discharge strength.

The increase in burst area gain may reflect a compensatory response. Baseline MSNA increases with age, potentially limiting capacity for further burst recruitment during pressure falls. Under such conditions, modulation of burst strength may help preserve sympathetic support of arterial pressure when recruitment capacity is constrained. This interpretation is consistent with those of Hart et al. (2011), who reported higher rising pressure gain in individuals with high baseline MSNA due to limited capacity for sympathoexcitation, and D'Souza et al. (2022), who reported that older adults exhibited preserved integrated MSNA baroreflex gain, yet action‑potential‐level analyses revealed enhanced central coding through increased gain of medium‑sized sympathetic discharge clusters. These were interpreted as selective amplification within the neural component of the baroreflex to compensate for reduced peripheral responsiveness to MSNA.

### 4.3 Mechanistic interpretation

Although the present findings point towards altered neural contributions, the underlying physiological mechanisms remain uncertain. Hysteresis of baroreflex responses may be related, at least in part, to intrinsic properties of the baroreceptors, which exhibit direction dependent differences in threshold and firing frequency in animal models (Angell‐James, 1971; Chapleau & Abboud, 1987). However, preservation of mechanical component gain does not necessarily imply preserved afferent signalling. Baroreceptor afferent output can change independently of the pressure–diameter relationship, resulting in lower firing rates and higher activation thresholds (Chapleau et al., 1989). To our knowledge, no study has examined age‐related changes in afferent hysteresis.

Beyond the afferent limb, broader evidence supports consideration of several potential neural mechanisms. Advancing age is associated with structural and functional remodelling within central autonomic circuits (Chapleau et al., 1995) and increased noradrenaline turnover (Esler et al., 2002), either of which could influence basal sympathetic or dynamic reflex responsiveness. Animal studies have identified discrete brainstem regions that govern timing and patterning of sympathetic discharge (Mueller et al., 2017), while human neuroimaging studies implicate the same regions in baroreflex control (Kramer et al., 2014). It is therefore plausible that attenuation of hysteresis in middle‐aged men reflects altered central autonomic processing, reduced reserve for sympathetic burst recruitment associated with elevated resting sympathetic activity or changes in sympathetic burst gating. While these possibilities are consistent with the present findings, they were not directly assessed and remain speculative.

Taken together, the available evidence and the present findings suggest that neural mechanisms, rather than mechanical impairment alone, may contribute to age‑related attenuation of neural hysteresis and, consequently, upward resetting of resting sympathetic outflow observed during midlife (Taylor & Tan, 2014).

### 4.4 Physiological implications

Regardless of the underlying mechanism, mid‐life attenuation of directional weighting within the sympathetic baroreflex may impair arterial pressure regulation during dynamic physiological challenges. Directional modulation of sympathetic outflow is thought to facilitate rapid baroreflex responses during transient pressure changes; consequently, reduced hysteresis may weaken baroreflex buffering during dynamic conditions such as standing or exercise.

In young men, small increases in arterial pressure from lower diastolic pressures elicited an immediate suppression of burst initiation, consistent with rapid baroreflex sympathoinhibition that is not fully captured by gain estimates alone. In contrast, middle‐aged men showed similar stimulus–response relations during rising and falling pressure, resulting in increased rising pressure gain and a loss of directional asymmetry of burst probability. This convergence of directional responses manifests as a loss of baroreflex hysteresis, rather than enhanced inhibitory control.

Accordingly, interpretation based solely on gain may misrepresent age‐related differences in autonomic control, if operating point, pressure range and recruitment limits are not considered. Under such conditions, preserved hysteresis in burst strength may help maintain directional modulation of sympathetic outflow when burst recruitment becomes less directionally sensitive and may represent recalibration of sympathetic baroreflex control in midlife.

### 4.5 Methodological considerations and limitations

Methodological differences between spontaneous analyses and pharmacological perturbation approaches should be considered when comparing results across studies. Phenylephrine administration during the modified Oxford protocol can abruptly suppress MSNA, limiting available data points for slope estimation, whereas spontaneous methods sample narrower pressure ranges but increase data density and physiological relevance. Importantly, baroreflex gain derived from pharmacological and spontaneous assessments is not equivalent (Rudas et al., 1999) and large imposed pressure excursions may reduce apparent gain in young adults (Hissen et al., 2018). In addition, vasoactive agents may influence central autonomic regulation directly. Given the established role of nitric oxide in modulating central sympathetic outflow (Hogan et al., 1999; Kienbaum & Peters, 2004), nitrovasodilator infusion may alter reflex characteristics through central as well as peripheral mechanisms – effects absent in spontaneous analyses.

Further limitations merit consideration. The study was cross‐sectional and restricted to men, precluding conclusions regarding sex‐related differences. Mechanical assessment relied on tracking common carotid artery diameter as a surrogate for barosensory deformation at the carotid sinus, acknowledging that regional differences in geometry and compliance may exist and these measures may not fully capture deformation at the baroreceptor site. Mechanical assessment also did not include direct measures of aortic diameter. In this context, carotid and aortic afferent signals converge centrally and cannot be readily separated in humans, limiting the extent to which regional contributions can be distinguished. Finally, while our findings are consistent with altered neural contributions to age‐related differences in baroreflex hysteresis, direct assessment of central mechanisms (e.g. neuroimaging) was not performed, and the present study cannot identify the specific site(s) within the neural arm of the baroreflex responsible for these differences. These methodological considerations highlight the need for future studies integrating multi‑site barosensory assessment and direct measures of central autonomic function to further clarify the mechanisms underlying age‑related changes in sympathetic baroreflex control.

### 4.6 Conclusion and perspectives

In healthy middle‐aged men, spontaneous vascular sympathetic baroreflex function is characterized by preserved mechanical gain but loss of hysteresis in burst initiation. There is evidence of burst strength hysteresis in young and middle‐aged men. By resolving the spontaneous vascular sympathetic baroreflex into its mechanical and neural components, this study adds to our knowledge on how age‑related loss of hysteresis reflects altered neural contributions under physiological conditions, providing new insight into mechanisms of blood pressure regulation at rest.

## AUTHOR CONTRIBUTIONS

Conception or design of the work: Guto Wyn Hughes, Denis J. Wakeham, Christopher J. A. Pugh, Rachel N. Lord, Jonathan P. Moore. Preparation for the work, acquisition, analysis or interpretation of data: all authors. Drafting the manuscript: Guto Wyn Hughes and Jonathan P. Moore. Critically revising for important intellectual content: all authors. All authors have read and approved the final version of this manuscript and agree to be accountable for all aspects of the work in ensuring that questions related to the accuracy or integrity of any part of the work are appropriately investigated and resolved. All persons designated as authors qualify for authorship, and all those who qualify for authorship are listed.

## CONFLICT OF INTEREST

None declared.

## GENERATIVE AI STATEMENT

Microsoft Copilot (GPT‐4 model) was used as an editorial assistant to review the structural organization of the manuscript and to check compliance with journal formatting guidelines. No AI tool was used for data analysis, interpretation of results or content generation. The authors maintained full intellectual control over the content, writing and interpretation of data.

## Data Availability

The data that support the findings of this study are available from the corresponding author upon reasonable request.
